# Integrated Quality Prediction Model for Food Quality Management Based on *E. coli* in Shared Kitchens

**DOI:** 10.3390/foods13244065

**Published:** 2024-12-17

**Authors:** Taeyeoun Roh, Youngchul Song, Byungun Yoon

**Affiliations:** Department of Industrial & Systems Engineering, Dongguk University, Seoul 04620, Republic of Korea; arongi1320@naver.com (T.R.); syc5002@naver.com (Y.S.)

**Keywords:** cloud kitchen, shared kitchen, food quality, quality prediction

## Abstract

Shared kitchens have a lower entry barrier than traditional kitchens, which generally require a significant initial investment, and have thus attracted attention as the most realistic new business model for restaurants in the sharing economy. The restaurant industry is founded on ensuring the safety of the food it serves in order to prevent the spread of foodborne diseases within the community, so strict quality control is essential. Existing food quality management typically employs continuous quality assistance, which is difficult to apply to the highly volatile shared kitchen environment and its various stakeholders. Therefore, in this study, a predictive model for managing food quality that can monitor volatility using quantitative indicators, especially microbial counts, is proposed. Stakeholder- and quality-related factors associated with shared kitchens are first defined, then a modified Gompertz growth curve and the transfer rate equation are used to quantify them. The proposed model, utilizing *E. coli* as a practical indicator for easily measuring changes in general environments, can be used to systematically manage food quality within the shared kitchen industry, thus supporting the establishment of this new business model.

## 1. Introduction

Shared kitchens, which are a space that provides cooking facilities that can be used jointly by several restaurants, have recently emerged as a solution to the instability in the restaurant industry caused by the COVID-19 pandemic, the rise in delivery and take-out food services, and the increase in real estate costs [1]. It has also been argued that they are the most realistic business model for the sharing economy because they minimize overlapping resources and maximize resource efficiency [2,3]. Unlike traditional restaurants, which require a large upfront investment and a business model based on commercial analysis, shared kitchens are widely used by startups and individual entrepreneurs to enter the market quickly because they can flexibly respond to changes in the business environment by adjusting the kitchen size, menu, and number of employees. Similar to the franchise model, shared kitchens can be used as a test bed for business expansion, such as by launching delivery-specific or event stores.

In the states of New York, Georgia, and Chicago in the United States, shared kitchens are already managed by the Department of Public Health or the Department of Agriculture [4]. This is a safe business form that allows government-led management and the monitoring of shared kitchens from a government-supervisor perspective. Because all of the typical processes of a restaurant, such as raw material supply, cooking, storage, and delivery, are carried out within shared kitchens, food safety and quality indicators can be measured and monitored at each stage. If a company in a shared kitchen is replaced by another, existing measurement and monitoring strategies can be maintained without the need for new outlays such as new sensors and monitoring personnel. In addition, by managing the quality control history for each dish, a database for the quality control of food can be established and used for subsequent research.

Food quality management requires quality control of the ingredients involved and environmental management when preparing, cooking, and serving the food. The quality of food items is associated with their color, taste, aroma, and texture, while environmental management targets the levels of microorganisms in the finished product and other materials in the restaurant related to the health and safety of the consumer. Environmental management also involves analyzing potential hazards in the food production process, selecting management points, and continuously monitoring them. Therefore, various standards and models have been proposed and implemented for food quality management. A widely adopted guideline related to food quality management is HACCP [5,6]. HACCP is a risk-based management system designed to identify contamination factors and prevent risks throughout the food supply chain, from raw materials to consumption. Similarly, ISO 22000 [7] is an international standard that applies across the entire food value chain and is used as a certification to ensure food safety.

Existing food quality management generally involves continuous quality assurance, where quality is consistently monitored and maintained through the quantitative/qualitative certification of the cooking environment. However, shared kitchens cannot guarantee food quality if the changeover between restaurants is rapid and they differ greatly in their menu and operating processes. In addition, a shared kitchen should have cookware for regular dishes, rather than specific dishes, from the standpoint of the shared kitchen’s operator. In order to ensure the quality and safety of food produced in a shared kitchen, a safety and quality control process for the cooking environment is essential [4,8]. Therefore, this study proposes a predictive quality management model tailored to shared kitchens based on quantitative data that is in alignment with the volatility of the shared kitchen industry and the characteristics of the various stakeholders involved.

## 2. Background

### 2.1. Shared Kitchens

Shared kitchens are a component of the sharing economy, where individuals share assets, resources, and services. By connecting those who wish to share resources with those who wish to use them, money and other resources can be saved [9]. The sharing economy can be broadly categorized based on the type of shared resources involved, with notable examples including ride-sharing (e.g., Uber), accommodation sharing (e.g., Airbnb), and office sharing (e.g., WeWork). Shared kitchens involve the sharing of kitchen spaces, constituting a major component of the food service industry, but also the sharing of cooking equipment such as ovens, which are often difficult to purchase and maintain, thus lowering the entry barriers for new food service businesses.

Shared kitchens can be classified as either open or individual shared kitchens [10,11]. Open shared kitchens allow multiple businesses to use the kitchen space simultaneously, sharing all the cookware, such as tables, refrigerators, and cutting boards. In contrast, individual shared kitchens divide the kitchen space into separate, independent areas, each equipped with its own cookware, allowing a single business to exclusively use the space. Depending on the contract type, a business can also secure long-term access to the space under a rental agreement. From a business perspective, shared kitchens can be categorized into five types (Table 1). Franchise operators typically run their own shared kitchens under types 1 and 5. Type 3 shared kitchens are widely adopted in densely populated areas, while the government (or local municipalities) utilizes type 2 and 4 shared kitchens for regional events, such as festivals, as a form of social service.

The quality of the food produced in shared kitchens not only directly affects customers’ health but also can serve as a medium for the proliferation of parasites and viruses, making strict quality control essential. In the U.S., individual states or cities impose specific regulations governing the environment and food quality management in shared kitchens. These regulations often include strict restrictions, such as limiting the types of food that can be produced and sold in shared kitchens or restricting certain processing methods.

Food quality can affect not only the individual consuming it but also others in the community because it can cause food poisoning and transmission. Therefore, strict quality control, cause analysis, and the prevention of contamination are essential [13]. Factors that affect food quality can be categorized into two main areas: the cooking environment and the food being prepared. In the event of an issue, it is crucial to identify who is responsible for managing the risk. Therefore, when using a shared kitchen, it is important to clearly define and specify the roles and responsibilities of the shared kitchen’s operator and the restaurant. In South Korea, the roles of the operator and the user are strictly distinguished based on the business model of the shared kitchen, and a formalized response process is in place to address any issues that may arise.

### 2.2. Food Quality Management Models

Food quality management models typically have three key objectives: consumer safety, regulatory compliance, and consistent quality. Food has a direct impact on the health of customers, either through direct ingestion or as a vector for viruses. Therefore, quality management models aim to preemptively avoid these risks [14]. Secondly, these models are used to ensure compliance with regulations at national, state, and city levels. Because the factors that can be checked at the final food product stage differ from those during the manufacturing stage, comprehensive quality management across the entire food manufacturing process is necessary. For example, in the case of fully cooked meat, microorganisms may have been eliminated during the heating process; however, microorganisms might have already proliferated prior to heating. Alternatively, microbial growth during storage or transportation after cooking could result in a situation where the food is safe immediately after heating but its quality cannot be guaranteed by the time it is consumed. Thus, models that allow for quality management at each stage of the food manufacturing process are employed [4,5].

Cooking methods utilizing quality management models approved by the government ensure the quality of food, allowing consumers to purchase safe products. Thirdly, these models help maintain consistent quality by controlling the environmental factors that influence food, such as temperature, humidity, and sanitary conditions, from the supply of raw materials to the delivery of the final product. Quality management models quantitatively measure these environmental factors during the food manufacturing process, identifying and addressing any deviations, thus ensuring consistent quality [5,15,16].

Quality management models can be categorized into standard-certification models, which include checklists for procedures, processes, and control points, and predictive models, which utilize quantitative data and formulas. A representative example of a standard-certification model is HACCP, which identifies and focuses on managing critical control points through hazard analysis, a strategy first developed by NASA in the 1960s [17]. HACCP is a preventive hygiene management system that systematically identifies and manages potential hazards, from the delivery of raw materials to the point before the customer consumes the product. HACCP provides benefits for consumers, the government, and the food industry, which has led to its widespread adoption in this industry. Other standard-certification systems in use worldwide are listed in Table 2.

Quality prediction models can be divided into those that predict the actual number of microorganisms present in food and those that predict quantified metrics related to food quality and the expiration date [18,19]. Microbial prediction models for food include the Gompertz model, microbial transfer rate equations, and equations utilizing the D-value. These models predict changes in the number of microorganisms over time based on various factors, starting from the initial microbial count. In terms of food quality and the expiration date, prediction models include the Arrhenius equation-based food prediction model and food quality models based on food quality indicators. The standard equations for these models are presented in Table 3.

#### 2.2.1. Microbial Growth During Storage

During the storage of food ingredients, the characteristics of the ingredients, the temperature and humidity of the storage location, and other environmental factors influence microbial growth. Therefore, microbial prediction models that take these factors into account have been actively researched. An example of this is the modified Gompertz model, which is widely used to determine the growth and inactivation of microorganisms. The Gompertz model dictates that mortality rates increase exponentially during a specific period. Based on this, Equation (7) is used to calculate the total population over time:

(7) $$y=d^{g^{t}}$$

where *y* represents the total population at time *t*, and *d* and *g* are model constants [24].

Given its usefulness in explaining microbial growth in biotechnology, various modified Gompertz models have been proposed to analyze the growth patterns of microorganisms [25]. Rogers [26] proposed a nonlinear growth model that accounts for the initial population and gradually decreases over time, while Zwietering [27] considered characteristics such as the maximum growth rate and lag time, making the graph intuitively understandable. The authors of [20] suggested a growth curve based on the Gompertz model that could more accurately predict the number of microorganisms (Equation (1)). This curve utilizes the maximum growth rate, the time at which the maximum growth rate is recorded, and the difference between the initial and maximum values of the microorganisms, characterized by an S-curve [20]. These various modified forms of the Gompertz model have been selectively applied depending on the experimental environment and the characteristics of the food materials involved, such as vegetables and chicken [25,28,29].

The Baranyi model is also based on an S-curve for microorganisms [21] (Equation (2)). Using an adjustment function (*A*(*t*)), the model assesses the adaptation of microorganisms to their environment, which is determined by λ. The larger the λ, the more difficulty microorganisms have in initially adapting to the environment. Consequently, during the initial adaptation phase, the growth of microorganisms is minimal. After a certain period, once the microorganisms have adapted to the environment, *A*(*t*) increases linearly. During this phase, the growth rate of the microorganisms is at its peak. At this point, the maximum growth rate is determined by μ. In the subsequent stationary phase, *A*(*t*) does not change significantly over time, and microbial growth is limited by the logarithmic term, eventually reaching saturation.

The Baranyi model has been used as a model to predict microbial contamination in vegetables [30,31] and to estimate the number of microorganisms in seafood and meat, meaning that it is applicable to a wide range of food [32,33]. Both the modified Gompertz and Baranyi models have been successfully fitted in studies predicting microbial growth curves, with most research achieving an *R*^2^ exceeding 0.9 [25,34,35].

#### 2.2.2. Equations Related to Microbial Inactivation Curves During Heating

During cooking, most microorganisms that can cause food poisoning are inactivated due to the high-temperature environment. Therefore, methods for predicting microbial counts that can account for thermal inactivation during the cooking process are currently being studied. The D-value, as presented in the Laboratory Manual for Food Canners and Processors, is defined as the time required to reduce the microbial count by 90% (Equation (3)). This concept serves as a tool for predicting microbial inactivation, assuming that the change in microbial count follows a first-order polynomial. Once the D-value is known for a specific reference temperature, the D-value at other temperatures can be predicted using the Z-value (Equation (4)), which represents the change in temperature required to cause a tenfold change in the D-value [36]. The microbial count after time *t* decreases more rapidly as the temperature increases, leading to a lower D-value, whereas a decrease in temperature results in a higher D-value, slowing the rate of microbial inactivation. By utilizing the relationship between the D- and Z-values, thermal inactivation processes can be predicted using simple equations, making this a fundamental model for the prediction of microbial inactivation [22,37,38,39,40]. These indicators are used as scientific measures to determine the necessary heat levels and time required to ensure product safety [41,42].

The Weibull model is a statistical model that represents microbial inactivation by considering biological variation related to thermal inactivation (Equations (5) and (6)). The Weibull model expresses the probability of survival at a specific time *t* depending on the scale parameter α and the shape parameter β. Thus, the microbial count after time *t* is predicted by multiplying the initial microbial count by the survival probability function. This model can take various forms depending on its parameters: α determines the time at which the count decreases, with a larger α resulting in a more rapid reduction in the microbial count; β determines the shape of the curve. If β is less than 1, the curve is concave downward, indicating that microorganisms die off quickly at first, but the rate of inactivation slows over time. Conversely, if β is greater than 1, the curve is concave upward [23,37,43]. Therefore, the Weibull model has been used in studies on microbial inactivation in food items because it allows for the development of survival curves tailored to the specific survival characteristics of microorganisms in different food materials [44,45].

#### 2.2.3. Equations Related to Predicting Microbial Counts During Cleaning, Pre-Processing, and Non-Thermal Processing

There has been no specific research conducted on the development of equations for the prediction of microbial counts related to the cleaning of food materials in the cooking process, despite there being a clear difference in microbial counts due to cleaning. Research results indicate that the microbial count on food ingredients decreases after cleaning with water [46,47], highlighting the need for this step to be included in integrated microbial count prediction models for the cooking process. In particular, the reduction rate due to cleaning can be experimentally determined and employed as a multiplier for the microbial count.

In the cooking process, various types of cookware are employed for the handling, processing, and cooking of food. During this process, microorganisms present on the cooking tools and the hands of the chef can be transferred to the food ingredients. However, the transfer rates between food ingredients and cooking tools vary significantly, affecting the risk of cross-contamination [14]. It is thus required to carefully consider cross-contamination during the non-heating food processing stages in order to accurately predict microbial counts. Previous studies have attempted to quantify transfer rates to account for cross-contamination and used this information to assess and manage the risks involved [48]. This study thus proposes an integrated quality prediction model tailored to various forms of shared kitchens, particularly those designed for delivery-based business models, that also accounts for potential cross-contamination between food handlers and cookware.

## 3. Integrated Quality Prediction Model for Shared Kitchens

This study proposes a model that can predict the quality of food produced in the cooking environment of shared kitchens based on controllable environmental factors. This prediction model uses quantitative data for the factors discussed in the previous section to determine the levels of harmful microorganisms in prepared food items as a quality indicator. To ensure that the proposed prediction model can be used in various shared kitchen environments, this study develops it in three stages. First, the standard cooking process in shared kitchens is defined. After that, non-heated foods that are susceptible to microbial contamination are selected and their cooking process is established. Finally, a prediction model that is most suitable for this process is established and tested.

### 3.1. Customized Quality Prediction Model for the Cooking Environment in a Shared Kitchen

The proposed integrated quality prediction model for shared kitchens aims to prevent safety risks from occurring. To this end, it is necessary to identify which factors impact food safety and how they can be managed. This is complicated by the fact that various stakeholders participate in a shared kitchen, and quality issues can arise due to various operational and environmental factors related to these stakeholders. Operational factors relate to human behavior and the kitchen rules involved in the management and cooking process, including cooking time, the hygiene management of cookware, transportation, and distribution management. Environmental factors directly impact food quality and include temperature, humidity, and air pollution levels (e.g., fine dust). These factors affecting the food quality associated with each stakeholder in a shared kitchen are summarized in Table 4.

Grocery delivery transports the food raw materials to the restaurant in the shared kitchen and requires an optional delivery schedule for multiple customers, though traffic issues can affect the quality of the food raw materials. The operator provides the space for the shared kitchen that the restaurant uses and directly manages the cleanliness of the cookware, such as knives and cutting boards, used by the restaurants, which can affect food quality by managing the transfer of microorganisms between utensils and food. The restaurant uses the shared kitchen to prepare, cook, and assemble the raw materials provided by the grocery delivery. At this time, the food quality is affected by the condition of the food materials and the cooking skills of the restaurant’s chefs. For example, if fresh materials are not used or if materials that quickly spoil at room temperature are not carefully managed, safety issues can arise. When cooking and assembly are complete in a shared kitchen, delivery and the destination are often managed through a delivery platform. The delivery platform calculates the time required to prepare the ordered food, receives the customer’s address, and allocates the delivery to the optimal delivery person, who then agrees to accept the request. This process, combined with the actual movement time, which depends on traffic conditions, dictates how long it takes before the customer receives the food. Temperature, humidity, and air quality affect all stakeholders in a shared kitchen, who use various methods, such as a refrigerated delivery truck, shared kitchen dehumidifiers, and air purifiers, to ameliorate the negative effects of these environmental factors.

In general, operational factors can be divided into delivery-related and cooking-related factors, while environmental factors affect all factors. Delivery is closely related to factors that cannot be directly controlled by the shared kitchen, such as the technology used by the delivery platform and regional characteristics. Therefore, this study proposes a prediction model for food quality that focuses on temperature and time, which can be controlled by shared kitchens. Therefore, this study proposes a prediction model for food quality that focuses on temperature and time, which can be controlled by shared kitchens. This focus on temperature allows various types of cooking processes to be considered, such as immediate meals, packaging, storage, and delivery after cooking and assembly are complete.

### 3.2. Quality Prediction Model for Non-Heated Food

This study proceeds with clarifying the terminology. Cooking refers to all actions that perform cooking, which can be divided into heated and non-heated methods depending on temperature. A shared kitchen can be employed to produce cooked food, non-heated food, and/or mixed food, depending on whether or not the food ingredients involved are heated to high temperatures for cooking. Heated food is raised to very high temperatures, such as through boiling, steaming, or grilling, and includes all dishes that are directly baked in a pan or oven or heated indirectly using water or boiling oil. In contrast, non-heated food is generally washed and processed at low temperatures. Dishes such as salad and sashimi are thus considered non-heated foods for the purposes of the proposed model. Mixed food generally combines heated and non-heated food items together, such as sandwiches (e.g., ham + vegetables + bread) and tacos (bread/shell + meat + raw vegetables + cheese).

The microorganisms used as a quality indicator in this study are inactivated when heated to an appropriate temperature and are generated by the decomposition of the food components due to storage or during reheating after cooking [49]. In addition, when meat is used in a dish, the time required to inactivate the microorganisms present depends on the type of meat, the heating type, and the type of cookware, and research has been conducted to determine the optimal cooking conditions for different types of meat [22,50,51]. The purpose of this study is to propose a quality prediction model in a shared kitchen environment that can be used universally, with the specific target of non-heated food, to which equations can be applied for the simple food materials involved. Udon noodles, wet rice noodles, and pre-cooked rice are produced in external facilities rather than in shared kitchens and are delivered to shared kitchens in a vacuum-sealed state. In shared kitchens, only simple cooking processes, such as heating and combining, are performed, and the overall quality is more significantly influenced by the state of the food prior to delivery. Therefore, these items are excluded from the scope of this study.

### 3.3. Standard Food Cooking Process and Model Design for Non-Heated Food

This study proposes a standard food cooking process for non-heated food consisting of five stages (Table 5). The environmental and operational factors associated with each step are expressed as quantitative equations using a Gibson–Gompertz growth curve and the microbial transmission equation. Both equations can be calculated based on the microorganism levels measured using simple experiments and can predict microbial levels over time and according to temperature.

In a shared kitchen, the restaurant secures the necessary food materials and then cleans, pre-processes, and stores them. When a customer order is received, the restaurant removes the food from storage and starts the non-heated processing process, preparing and assembling the dish for delivery. During this stage, of the various influential environmental factors, food materials are most strongly affected by temperature [52]. Although methods to suppress microbial growth using ozone [53] and low-temperature plasma [54] have been proposed, their application in a dynamic shared kitchen environment requiring real-time measurement has been excluded. Therefore, these methods are not considered in this study.

**Figure 1:**
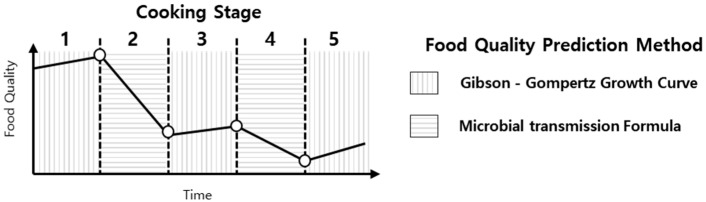
Overview of the proposed food quality prediction model.

In this study, the growth rate of microorganisms during the storage and serving/delivery stages is predicted using C, B, and M, the parameters used in Gibson’s modified Gompertz model, which affects the temperature and the bacterial growth on food materials [20]. In the cleaning/pre-processing and non-heated processing stages, the microbial transmission equation, which is affected by the cleanliness of the cookware in the shared kitchen, is used. The predictive model for the standard cooking process using the two equations is summarized in Figure 1. In practical scenarios, food packaging materials such as biodegradable plastics may be used and could have unique transfer rates. However, for the purposes of this study, it is assumed that the containers used during delivery do not contribute to microbial growth.

## 4. Development of a Prediction Model for Egg Salad Quality

In this study, a prediction model for egg salad quality according to the established standard cooking process is developed, thus enabling response strategies to be established for shared kitchens. Egg salad is prepared by combining boiled egg, which is pre-cooked by another company and delivered to the restaurant, and lettuce with various dressings to suit the customer’s preference. Egg salad can be prepared without any special cooking tools and can be served directly to the customer on-site or delivered, so it has the potential to be made in all shared kitchen models. Because the dressing is added in the last step before the customer receives the dish, this study builds a quality prediction model targeting the boiled egg and lettuce ingredients. In this study, the number of microorganisms is identified for *Escherichia coli* (*E. coli*), where the standards of public institutions are specified among several microorganisms.

*E. coli* was selected as a microorganism to predict food quality, and a quality prediction model was constructed. Listeria, Salmonella, and Campylobacter, which are foodborne pathogens, are capable of growing across a wide range of temperatures and pH values and exhibit specific behaviors depending on the protein content of food. Moreover, they can be eliminated by factors outside controlled conditions. However, *E. coli* demonstrates a relatively predictable growth pattern within typical growth ranges, making it a reliable indicator microorganism for assessing food hygiene [55,56].

Egg salad is a ready-to-eat food, and the Philippines Food and Drug Administration has suggested Escherichia coli levels of less than 100 CFU/g per 1 g as the food quality standard for this food type [57]. The proposed prediction model thus complies with this standard and sets the quality threshold at below 100 CFU/g per 1 g at the completion of non-heated processing before the addition of the dressing. To measure the inherent levels of microorganisms, the CFU/cm^2^ unit is typically used. However, in this study, the CFU/g unit was utilized to quantify the microbial levels in food. During the process of measuring microbial levels per unit area (cm^2^) of food, changes in the physical properties of the food may lead to variations in the microorganism count. Therefore, this study measured microbial levels using the CFU/g unit.

### 4.1. Stages 1, 3, and 5—Fitting of the Gibson–Gompertz Growth Curve

**Figure 2:**
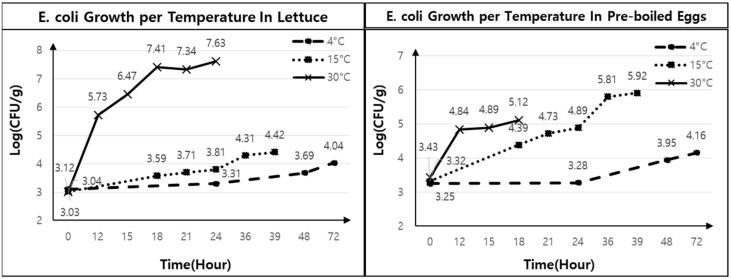
*E. coli* growth curves for lettuce (**left**) and pre-boiled eggs (**right**) according to the temperature.

**Figure 3:**
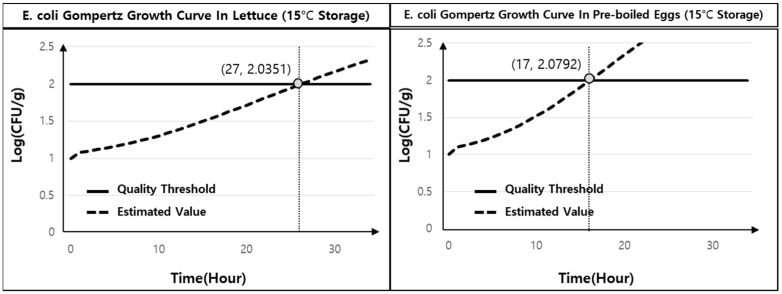
*E. coli* growth curves for lettuce (**left**) and pre-boiled eggs (**right**) stored at 15 °C.

Pre-boiled eggs and lettuce follow a standard cooking process and are combined before being served to consumers. In order to fit the Gompertz growth curve, which can predict the change in food quality during storage (Stages 1 and 3) and serving (Stage 5), the change in *E. coli* levels over time was measured at temperatures of 4, 15, and 30 °C for the pre-boiled egg and lettuce (Figure 2). The parameters required to calculate the Gompertz growth curve are presented in Table 6, with the calculations conducted using GraphPad Prism 10. The R2 value of the fitted result was calculated as 0.984 for lettuce and 0.840 for boiled eggs, confirming that the fitting was completed well. Assuming that the initial *E. coli* density was 10 CFU/g, the fitted growth curve indicated that, when stored at room temperature (15 °C), the quality of lettuce was found to exceed the threshold value when stored for more than 27 h, while the pre-boiled egg was found to exceed the threshold after 17 h (Figure 3).

### 4.2. Stages 2 and 4—Development of a Prediction Equation for Food Quality Using the Cleaning and Transfer Rates

*E. coli* can be removed by physical cleaning utilizing water [58]. The pre-boiled egg and lettuce are washed with water, cut to the required size with a knife, and then stored. When an order is received, they are removed from storage, mixed in a bowl, and served to the customer. Stage 2 involves the use of water, hands, a knife, and a cutting board, while Stage 4 involves hands and a bowl.

To determine the effect of cleaning on *E. coli* levels in Stage 2, the average *E. coli* levels before and after cleaning were compared (Table 7). Subsequently, during Stages 2 and 4, the rate of transfer for *E. coli* present on the hands, knife, and cutting board to the lettuce and eggs was assessed (Table 8).

During Stage 2 cleaning and pre-processing, because the ingredients are taken out from storage, Stage 2’s equation is composed in the form of the cleaning ratio compared to Stage 4, as shown in Equations (8) and (9). Assuming that the initial amount of *E. coli* is 10 CFU/g, the levels of *E. coli* remaining in the lettuce and pre-boiled egg after Stages 2 and 4 can be predicted, as shown in Table 9.

(8) $$\left(E.\;coli\;levels\;after\;Stage\;2\right)=Initial\;E.\;coli\;levels\;before\;Stage\;2\times Cleaning\;rate+\;\sum ({E.\;coli\;levels\;on\;cookware}_{i}\times {Transfer\;rate}_{i})$$

(9) $$\left(E.\;coli\;levels\;after\;Stage\;4\right)=Initial\;E.\;coli\;levels\;before\;Stage\;4+\;\sum {(E.\;coli\;levels\;on\;cookware}_{i}\times {Transfer\;rate}_{i})$$

### 4.3. Integrated Prediction Model for Egg Salad Quality

**Figure 4:**
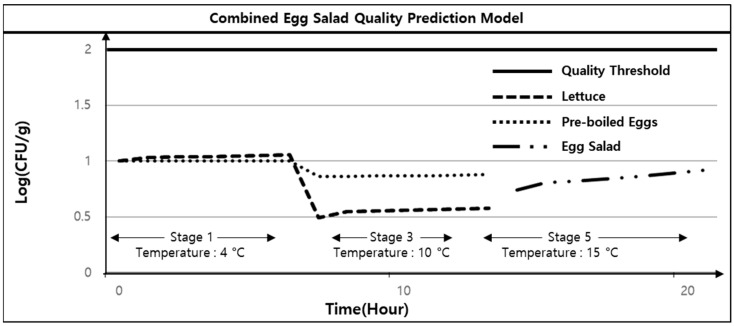
Combined prediction model for *E. coli* levels in egg salad.

According to the standard cooking processes proposed in this study, the lettuce and pre-boiled eggs are stored in Stage 1 and Stage 3, respectively, and in Stage 4, they are combined in one place. Even after mixing, it is assumed that the *E. coli* growth in the two ingredients follows the Gompertz equation, and it is assumed that the *E. coli* levels in the egg salad are in accordance with the mixing ratio of lettuce to pre-boiled egg. The temperatures in Stages 1, 3, and 5 were assumed to be 4, 10, and 15 °C, respectively, assuming low-temperature refrigerator storage, showcase storage, and room temperature, respectively, with Stages 1 and 3 set at 6 h each. The initial levels of *E. coli* on the cookware and hands were assumed to be 10 CFU/g. Based on these settings, the results of the proposed egg salad quality prediction model for Stages 1 to 5 are presented in Figure 4.

Assuming the initial levels of *E. coli* at all stages is 10 CFU/g, and *E. coli* growth follows the Gompertz equation in accordance with the temperatures established for each stage, the quality threshold should not be surpassed in the standard cooking process for egg salad.

## 5. Testing the Prediction Model for the Assessment of Food Quality

The quality prediction model proposed in this study can be used based on temperature and time, both of which affect the quality of food materials. Therefore, quality issues were predicted for cases where the temperature and time deviated from the standard cooking process, and response strategies were suggested.

### 5.1. Stage 1—Shared Kitchen and Shared Refrigerator Temperature

**Figure 5:**
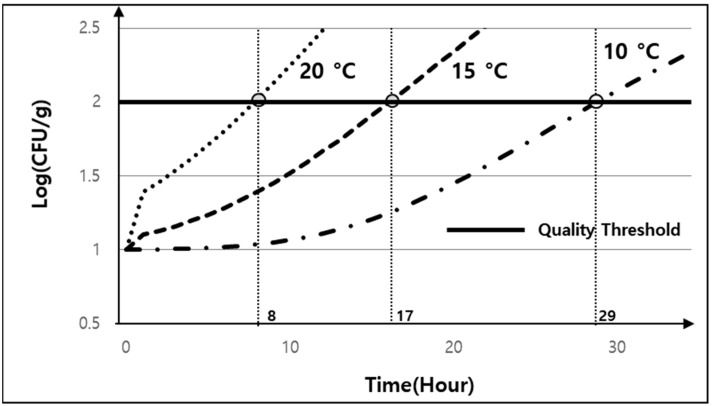
Change in *E. coli* numbers in pre-boiled eggs during Stage 1 storage according to the temperature.

Depending on the type of shared kitchen, several restaurants may share a refrigerator rather than using their own, and the optimal temperature for the refrigerator will differ depending on the food materials for each restaurant. According to the quality prediction model in Figure 4, because the levels of *E. coli* are highest in the initial storage stage, it is important to ensure that the levels of *E. coli* do not exceed the critical point at this stage. Figure 5 presents the change in *E. coli* levels for the pre-boiled egg according to the storage temperature during Stage 1 storage. When the temperatures for the shared kitchen external storage, shared kitchen internal storage, and showcase storage were 20, 15, and 10 °C, respectively, the quality threshold was exceeded after 8, 17, and 29 h, respectively. Based on this, the restaurant must perform Stage 2 cleaning and pre-processing within these times. In addition, the operator should inform the restaurants that the characteristics of the food ingredients used by them in the shared kitchen and ingredients that reach the quality threshold quickly should be stored in a shared refrigerator set at a lower temperature. In fact, when stored below 4 °C (i.e., low-temperature storage), the threshold value was not reached even after 48 h, so it was excluded from Figure 5.

### 5.2. Stage 3—Exposure to High Temperatures After Pre-Processing

**Figure 6:**
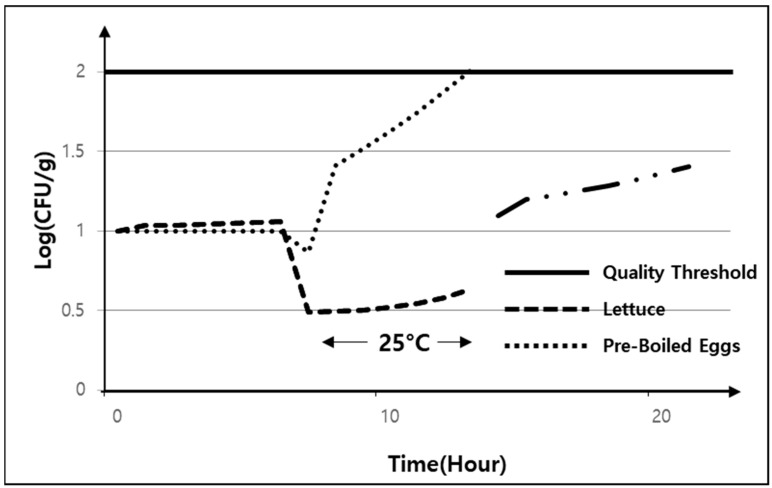
Effect of exposure to a high temperature (25°) on *E. coli* numbers after pre-processing.

If food is left at high temperatures due to carelessness after pre-processing, *E. coli* may rapidly increase depending on the food material. In Stage 2, the pre-boiled egg and lettuce are washed with water and pre-processed using cookware. During this process, if the cookware is not clean, *E. coli* may transfer to the food. Figure 6 presents the results when the lettuce and pre-boiled egg are left at a high temperature, showing that the number of *E. coli* rapidly increased in the pre-boiled egg. Therefore, restaurants must store food at low temperatures when the temperature of the pre-processing environment is high. Shared kitchen operators must distinguish between Stage 1 storage and Stage 3 storage so that restaurants can efficiently store pre-processed food materials and operate low-temperature refrigerators and open refrigerators with higher temperatures in accordance with the restaurant’s food supply plan while maintaining a stable set temperature.

### 5.3. Stage 5—Long-Term Exposure to High Temperatures

**Figure 7:**
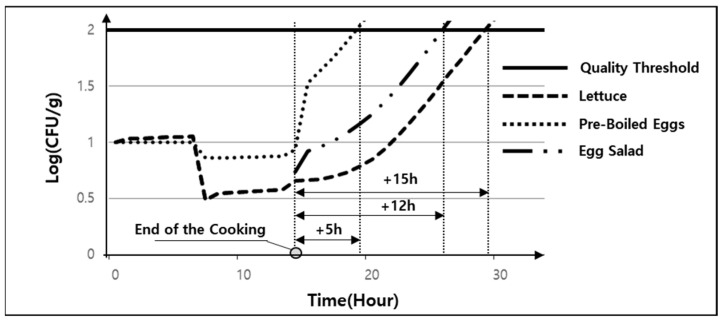
Change in *E. coli* numbers at 25 °C after cooking.

If displayed, delivered, and stored at high temperatures after cooking, the quality of the egg salad will exceed the *E. coli* threshold. When the quality of the egg salad is calculated, the quality threshold is not exceeded if consumed within 12 h, but when the quality is measured for pre-boiled eggs alone, the value is exceeded after 5 h (Figure 7). Considering the delivery and storage time and temperature, delivery companies should establish a cold supply chain, and restaurants that provide food should provide recommended intake times at high and low temperatures. The shared kitchen operator can select restaurants based on the time it takes to reach the quality threshold of ready-to-eat products. For example, *E. coli* levels can help operators determine whether certain food products require stricter hygiene protocols or cold chain management to ensure consumer safety. Also, because shared kitchens in the city can reach customers very quickly, fresh food restaurants serving sushi and oyster-based dishes can be allowed to use the shared kitchen.

### 5.4. Stage 2 to 5—Exposure to High Temperatures After Storage

**Figure 8:**
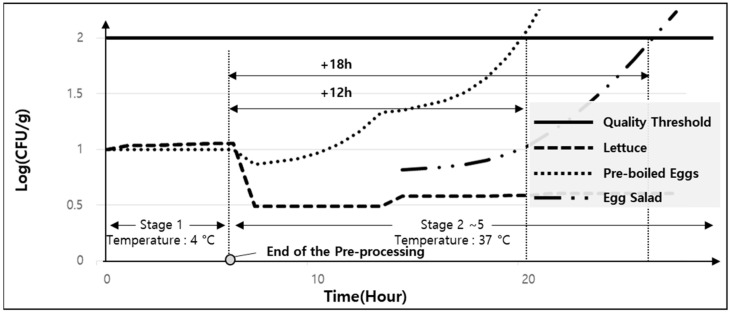
Change in *E. coli* numbers at 35 °C after pre-processing.

In shared environments such as food trucks and community potlucks, where external facilities are utilized, food can be prepared at an ambient temperature after the initial storage stage. This assumes that the pre-processed food is not returned to cold storage and a constant temperature is maintained, which can be applied to the prediction model. Figure 8 illustrates the quality changes in food prepared externally at 37 °C, a temperature ideal for *E. coli* growth. Due to its lack of protein, lettuce does not exhibit a rapid increase in microbial growth even at 37 °C. In contrast, boiled eggs show a significant increase in microbial levels following pre-processing, with the microbial count exceeding the threshold for egg salad after 18 h. However, for boiled eggs alone, the threshold is exceeded after 12 h. Based on these results, community potluck administrators are advised to serve egg salad only for breakfast or lunch. If storage is required, consumption should be limited to the same day and no later than dinner.

## 6. Conclusions

This study proposed a quality prediction model to manage food quality in the shared kitchen industry, which has been growing recently. The cooking process for a shared kitchen was analyzed, and the related stakeholders and the quality factors related to each stakeholder were analyzed. The environmental factors that affect food quality were then selected, and a standard cooking process consisting of five stages was established based on the characteristics of non-heated dishes. The Gompertz equation and the microbial transmission equation were then used to construct a quality prediction model reflecting the cooking process and environmental factors across the five stages. To test the prediction model proposed in this study, the levels of *E. coli* in egg salad were predicted based on processing stages 1, 3 and 5.

A quality prediction model has been proposed to reflect the unique characteristics of shared kitchens, which differ from traditional restaurants. In shared kitchens, the responsibility for quality is not concentrated in a single entity; instead, it is distributed across various stakeholders, making it distinct from conventional restaurants. Additionally, because multiple users share cooking tools within the kitchen, quality issues caused by these shared tools are more likely to arise. Thus, the study categorized the cooking environment of shared kitchens and clearly defined the variables that can be managed within the standard process. Through simulations under various conditions, potential quality issues were analyzed on a scenario basis. This model can serve as an effective management tool for restaurants using shared kitchens and the operators managing these spaces. It also ensures that consumers will receive food of stable quality produced in shared kitchens. In addition, this study used *E.coli*, a microorganism that can grow in a general environment, so that the quality of food can be predicted for various other microorganisms based on this model. For example, a sensor for measuring humidity can be placed in a shared kitchen environment, and it can be converted into a quality prediction model based on Salmonella bacteria according to humidity.

However, this study has a limitation in that the variables were simplified for the shared kitchen and cooking process. For example, *E. coli* levels can be influenced by various factors other than temperature, and the transfer of *E. coli* between food materials such as egg salad was not reflected. Similarly, various chemical interactions that may occur during the mixing process, depending on the characteristics of the food, were not considered. These limitations can be solved in two ways. First, it is possible to establish a more realistic growth curve for *E. coli* by adding other factors related to its growth as independent variables to the proposed quality prediction model. Second, the prediction model could be founded on more specialized cooking experiments to improve the accuracy of the food quality predictions.

The present study indicates that the most pressing problem facing shared kitchens is ensuring that quality control is at the same level as that in the traditional restaurant industry. In order to achieve this, it is suggested that the responsibility for quality management in shared kitchens be shared between stakeholders using quality prediction models based on relevant environmental variables. More sophisticated quality prediction models than that presented in this study, such as those that consider cooked food, will be possible in future research.

## Figures and Tables

**Table 1:** Business models for shared kitchens [12].

Business Model	Description
Incubating	In addition to kitchen space, the provision of startup support services such as menu development, culinary training, and marketing
Food hospitality	Equipped with kitchen facilities suitable for running a food service business and leased to users
Delivery	Multiple kitchen facilities are provided, allowing multiple businesses to occupy each space and use the kitchens primarily for delivery purposes
Delivery & food hospitality	Primarily intended for delivery but also offers a shared customer-facing area
Delivery & service	Primarily intended for delivery, with the shared kitchen operator providing consulting services such as marketing and culinary expertise

**Table 2:** Standard-certification systems in use globally.

Standard Certification	Definition and Characteristics
ISO22000	Food Safety Management Systems A food safety management system established by the International Organization for Standardization (ISO) including HACCP principles and integrating management system requirements
GMP	Good Manufacturing Practices Rules that define production and testing procedures to ensure the consistent quality and safety of food, including hygiene, equipment management, staff training, and quality control
IFS	International Featured Standards Reviews products and production processes to assess food producers’ ability to produce safe, informed, and high-quality products in accordance with legal requirements and customer specifications
SQF	Safe Quality Food SQF food safety regulations are designed to meet industrial, customer, and regulatory requirements for all sectors of the food supply chain, from farms to retail stores
BRC	British Retail Consortium A food safety and quality management standard developed by the British Retail Consortium setting safety, quality, and operational standards
FSMA	Food Safety Modernization Act Transforming the country’s food safety system by shifting the focus from responding to foodborne diseases to prevention

**Table 3:** Models used for the prediction of microbial levels and food quality.

**Model**	Description and Equations	
Gibson’s modified Gompertz [20]	Microbial predictions based on the maximum microbial count, lag time, and maximum microbial growth rate	
	$\log\left(y\right)=A+(Ce^{{-e}^{-B\left(t-M\right)}})$	(1)
	*y*: number of microorganisms at time *t*, *t*: time (h), *A*: initial number of microorganisms, *C*: difference between the maximum and the initial number of microorganisms, *B*: maximum growth rate of microorganisms, *M*: time at which the maximum growth rate is recorded	
Baranyi [21]	Predicting the microbial growth curve by assessing microbial adaptation to the environment	
	$y=y_{0}+\mu A\left(t\right)-\ln\left(1+\frac{e^{\mu A\left(t\right)}-1}{e^{y_{\max}-y_{0}}}\right)$	(2)
	*y*: number of microorganisms at time t, *y*_0_: initial number of microorganisms, *t*: time (h), *μ*: maximum microbial growth rate, *A*(*t*): environmental adaptation function, *y_max_*: maximum microbial count, λ: microbial lag phase constant	
D-value [22]	The time required to inactivate 90% (1 log CFU/g) of microorganisms at a specific temperature, assuming that the microbial inactivation rate is constant at a given temperature	
	$\log\left(\frac{y}{y_{0}}\right)=-\frac{t_{m}}{D}$	(3)
	$\log D=\log D_{\mathrm{ref}}-\frac{\left(T-T_{\mathrm{ref}}\right)}{z}$	(4)
	*y*: number of microorganisms at time *t_m_*, *y*_0_: initial number of microorganisms, *t_m_*: time (min), *D*: D-value at a specific temperature, *D_ref_*: D-value at the reference temperature, *T_ref_*: temperature at which the known D-value is measured, *z*: temperature change required to cause a tenfold change in the D-value	
Weibull [23]	Predicting the number of microorganisms through a function that represents the probability of microbial inactivation over time, assuming the relationship between microbial survival rate and time is not linear at a given temperature	
	$N_{t}=N_{0}\times S\left(t_{m}\right)$	(5)
	$S\left(t\right)=\exp\left(-{\left(\frac{t}{\alpha }\right)}^{\beta }\right)$	(6)
	*y*: number of microorganisms at time *t_m_*, *y*_0_: initial number of microorganisms, *t_m_*: time (min), *S*(*t_m_*): survival probability of microorganisms at time *t_m_*, α: scale parameter, β: shape parameter	

**Table 4:** Stakeholders and factors affecting food quality.

Business Model					Stakeholders	Factors Affecting Food Quality	
1	2	3	4	5		Operational	Environmental
O	O	O	O	O	Grocery delivery	Optimal delivery schedule, traffic issues	Temperature, humidity, air pollution
O	O	O	O	O	Shared kitchen	Kitchen cookware cleanliness	
O	O	O	O	O	Restaurant in a shared kitchen	Condition of food, cooking skills/processes	
		O	O	O	Delivery platform	Platform performance, optimal delivery system	
		O	O	O	Food delivery	Traffic issues	

Business models: 1: Incubating, 2: Food service, 3: Food delivery, 4: Food service & delivery, 5: Food delivery & consulting.

**Table 5:** Standard non-heat-related food cooking processes.

Process	Cooking Process	Factors Affecting Food Quality		Models
		Operational	Environmental	
1	Storage	Initial condition of food	Temperature	Gibson–Gompertz growth curve
2	Cleaning and pre-processing	Pre-processing skills/process, kitchen cookware cleanliness		Microbial transmission equation
3	Storage			Gibson–Gompertz growth curve
4	Non-heated processing	Processing skills/process, kitchen cookware cleanliness		Microbial transmission equation
5	Serving/delivery			Gibson–Gompertz growth curve

**Table 6:** C, B, and M quadratic polynomial parameters for lettuce and pre-boiled eggs and the fitting results.

	Lettuce			Pre-Boiled Egg		
	C	B	M	C	B	M
B_0_	1.4110	0.0461	54.6500	0.2424	0.1292	44.9100
B_1_	–0.0058	–0.0053	–2.8610	0.1572	–0.0068	–2.3070
B_2_	0.0038	0.0004	0.04553	0.0039	0.0001	0.0400
$R^{2}$	0.984			0.840		
RSS	1.165			27.870		
TSS	74.634			173.790		
SE	0.185			0.845		
MRE	3.333			7.55		

**Table 7:** Cleaning rate by food ingredient.

Lettuce			Pre-Boiled Egg		
*E. coli* Levels (CFU/mL)		Cleaning Rate (%)	*E. coli* Levels (CFU/mL)		Cleaning Rate (%)
Before	After		Before	After	
170,000	2600	98.47	440,000	80,400	81.72
140,000	3400	97.57	460,000	95,400	79.26
9000	2100	97.66	680,000	122,000	82.03
Average		97.90	Average		81.03

**Table 8:** *E. coli* transfer rate by food ingredient and source.

	Transfer Rate (%)			
	Hand	Knife	Cutting Board	Bowl
Lettuce	7.35	10.65	10.45	37.16
Pre-Boiled egg	10.93	18.89	24.07	37.78

**Table 9:** Estimated *E. coli* levels after Stages 2 and 4.

	*E. coli* Levels	
	Lettuce	Pre-Boiled Egg
Stage 2	7.35	10.65
Stage 4	10.93	18.89

## Data Availability

The original contributions presented in this study are included in the article. Further inquiries can be directed to the corresponding author.
